# Comparison of Product Carbon Footprint Protocols: Case Study on Medium-Density Fiberboard in China

**DOI:** 10.3390/ijerph15102060

**Published:** 2018-09-20

**Authors:** Shanshan Wang, Weifeng Wang, Hongqiang Yang

**Affiliations:** 1 College of Economics and Management, Nanjing Forestry University, No. 159, Longpan Road, Nanjing 210037, China; wssnjfu@outlook.com; 2 Research Center for Economics and Trade in Forest Products of the State Forestry Administration (SINO-RCETFOR), No. 159, Longpan Road, Nanjing 210037, China; 3 College of Biology and the Environment, Nanjing Forestry University, No. 159, Longpan Road, Nanjing 210037, China; wang.weifeng@njfu.edu.cn; 4 Co-Innovation Center for the Sustainable Forestry in Southern China, Nanjing Forestry University, No. 159, Longpan Road, Nanjing 210037, China; 5 Center for the Yangtze River Delta’s Socioeconomic Development, Nanjing University, No. 22 Hankou Road, Nanjing 210093, China

**Keywords:** life cycle assessment (LCA), greenhouse gas (GHG) emissions, carbon storage, publicly available specification (PAS) 2050, environmental hotspots

## Abstract

Carbon footprint (CF) analysis is widely used to quantify the greenhouse gas (GHG) emissions of a product during its life cycle. A number of protocols, such as Publicly Available Specification (PAS) 2050, GHG Protocol Product Standard (GHG Protocol), and ISO 14067 Carbon Footprint of Products (ISO 14067), have been developed for CF calculations. This study aims to compare the criteria and implications of the three protocols. The medium-density fiberboard (MDF) (functional unit: 1 m^3^) has been selected as a case study to illustrate this comparison. Different criteria, such as the life cycle stage included, cut-off criteria, biogenic carbon treatment, and other requirements, were discussed. A cradle-to-gate life cycle assessment (LCA) for MDF was conducted. The CF values were −667.75, −658.42, and 816.92 kg of carbon dioxide equivalent (CO_2_e) with PAS 2050, GHG protocol, and ISO 14067, respectively. The main reasons for the different results obtained were the application of different cut-off criteria, exclusion rules, and the treatment of carbon storage. A cradle-to-grave assessment (end-of-life scenarios: landfill and incineration) was also performed to identify opportunities for improving MDF production. A sensitivity analysis to assess the implications of different end-of-life disposals was conducted, indicating that landfill may be preferable from a GHG standpoint. The comparison of these three protocols provides insights for adopting appropriate methods to calculate GHG emissions for the MDF industry. A key finding is that for both LCA practitioners and policy-makers, PAS 2050 is preferentially recommended to assess the CF of MDF.

## 1. Introduction

The reduction of greenhouse gas (GHG) emissions has become a critical issue with the increasing focus on climate change. Carbon footprint (CF, tons of CO_2_e) has become a topic of broad and current interest to quantify the climate impact of products [1]. Owing to the need to quantify the life cycle carbon contributions of products, numerous methods have been developed for CF calculations [2,3]. In general, these methods follow three main protocols: Publicly Available Specification (PAS) 2050 published by the British Standards Institution (BSI) and revised in 2011 [4,5], GHG Protocol Product Standard (GHG Protocol) convened by the World Resources Institute (WRI) and the World Business Council for Sustainable Development (WBCSD) [6], and the latest, ISO 14067 Carbon Footprint of Products (ISO 14067) provided by the International Organization for Standardization (ISO) in 2012 [7]. PAS 2050 is the first CF accounting protocol at a product level. GHG Protocol was established based on ISO standards and the first version of PAS 2050. The three core protocols are based on ISO 14040 and ISO 14044 [8,9] on life cycle assessment (LCA).

Although the general principles of the preceding protocols mentioned are the same, the different criteria provided may lead to different results. By conceptually comparing the current methods and estimations [10,11], the results showed that concerns remain, despite the development and comprehensiveness of CF protocols. Existing disagreements in the calculation criteria and slight coherence are to be covered in CF calculations. Several comparisons on the CF accounting methods have been conducted by different products, such as biofuel [12], agricultural products [13,14], and forest products including office paper [15], poinsettia [16], and particleboard [2]. The use of different protocols leads to numerical differences in CF. The key aspects causing inconsistency originate from the system boundary, cut-off criteria, biogenic carbon treatment, allocation, and other requirements. Without coordination on these aspects, results may be biased.

Given that China has committed to decreasing its carbon dioxide (CO_2_) per unit of gross domestic production by 40–45% compared with that in 2005, numerous industries are actively pursuing the reduction of GHG emissions [17]. Regarding GHG emissions, forest products are better alternatives than other materials [18]. The role of the forestry sector in mitigating climate change is widely recognized, because forests absorb large amounts of carbon dioxide equivalent (CO_2_e) from the atmosphere. Through harvesting practices, stored CO_2_e can be transferred to wood products for extended periods, especially long-living products, such as wood-based panels [19]. Although wood emits less GHG compared with other materials, quantification of the environmental impacts during their life cycle is still necessary.

Medium-density fiberboard (MDF) (functional unit: m^3^) is currently produced and utilized worldwide. MDF represents approximately 88.77% of the fiberboard industry, with a total production of 59.04 million m^3^ in 2016. This amount is considerably higher than in Turkey (5.07 million m^3^/a), the world’s second largest country [20]. MDF is widely used in furniture, construction, packaging, flooring, and other industries, with the furniture industry consuming approximately 65% of the total production. In addition, China has become one of largest powers in trading MDF products. MDF is mainly exported to the United States, Saudi Arabia, and Canada and imported from New Zealand, Germany, Australia, and other countries. The demand for MDF in China remains strong, and the proportion of MDF in the wood-based panel industry is expected to increase further. However, the comprehensive energy consumption per unit of MDF in China is 4.8 times that of the average level in the world [21]. Moreover, MDF production yields relatively higher GHG emissions compared with other panels [22]. Thus, studies on improving the environmental profile of the MDF industry should be conducted. The energy consumption and environmental impact of MDF production were the focus of previous studies [23,24,25,26,27,28,29]. Despite the same method of LCA, researchers followed different protocols. With several competing protocols, determining the most suitable protocol for MDF and the wood-based panel industry is necessary.

This study aims to conduct a comprehensive CF analysis for MDF by comparing three general protocols, namely, PAS 2050, GHG Protocol, and ISO 14067. The goal is achieved by the following objectives: to recognize which aspects and criteria cause differences in the case study (cradle-to-gate) through comparisons and evaluate the CF of MDF based on PAS 2050 with end-of-life scenarios (cradle-to-grave). These three protocols were to be compared using the same manufacturing plant. The results have been applied to identify the main hotspots that provide the most contribution to environmental impacts. By considering the characteristics of wood production chains, this study provides insights for adopting appropriate methods to calculate GHG emissions for the MDF industry.

## 2. Methods

This study was conducted for a “state-of-the-art” MDF manufacturing company in China. The production system is in line with GB/T 11718−2009 Medium-Density Fiberboard, which is a national standard for China’s MDF. Since the start of the production, the annual production capacity reached 210,000 m^3^/a, which is at the forefront of the industry. We employed three protocols to calculate CF, namely PAS 2050, GHG Protocol, and ISO 14067. These protocols follow the life cycle thinking approach [30]. Therefore, two cases were evaluated: one from raw material extraction to MDF production (cradle-to-gate), the other from resource acquisition to the use and end-of-life stages (cradle-to-grave). The density of manufactured MDF is usually 726 kg/m^3^ with a moisture content of 30%.

CF is calculated by summing up the GHG emissions that are directly or indirectly attributed to the product during its life cycle [31,32]. Equations (1)–(3) illustrate how the GHG emissions of fuel and electricity are calculated based on the energy consumption and emission factors for China.

(1) $$E_{f,GHG}=FC_{f}\times EF_{f,GHG}\;$$

where *E_f,GHG_* is the GHG emissions from fuel combustion in the stationary sources (kg CO_2_e), *f* denotes the types of energy used, *FC_f_* is the consumption of energy *f* (MJ^−1^), and *EF_f,GHG_* is the emission factor for the energy *f* per GHG (kg CO_2_e MJ^−1^).

(2) $$\;E=a\left[Fuel_{a}\times EF_{a}\right]\;$$

where *E* is the GHG emissions from mobile sources (kg CO_2_e), *a* denotes the types of fuel used, *Fuel_a_* is the total use of fuel *a* (MJ), and *EF_a_* is GHG emissions from one MJ of fuel *a* (kg CO_2_e MJ^−1^).

(3) $$\;E_{e,GHG}=FC_{e}\times EF_{e,GHG}\;$$

where *E_e,GHG_* is the GHG emissions from electricity use in the stationary sources (kg CO_2_e), *FC_e_* is the consumption of purchases electricity *e* (kWh^−1^), and *EF_e,GHG_* is the emission factor for electricity *f* per GHG (kg CO_2_e kWh^−1^).

In the following subsections, the specifications and requirements for CF calculations provided in PAS 2050, GHG Protocol, and ISO 14067 were discussed to compare their differences. The goal of PAS 2050 and ISO 14067 is to initiate uniform methods to calculate CF, whereas the GHG Protocol aims to provide guidelines on accounting and reporting. An overview of the different aspects that affect the results are provided in Table 1.

An important characteristic of wood production chains is their multi-functionality, which addresses the allocation of the environmental performance between products and their co-products [33]. However, the surveyed company consumed branch woods, and no wood residues were collected from sawmills. In addition, the environmental burden in the production was totally allocated to the product, because no other co-products of significant mass (0.97%) were sold outside. This study used mass allocation; therefore, different allocation rules in analyzed protocols were not discussed.

### 2.1. System Boundary

#### 2.1.1. Life Cycle Stage Inclusion

**Figure 1:**
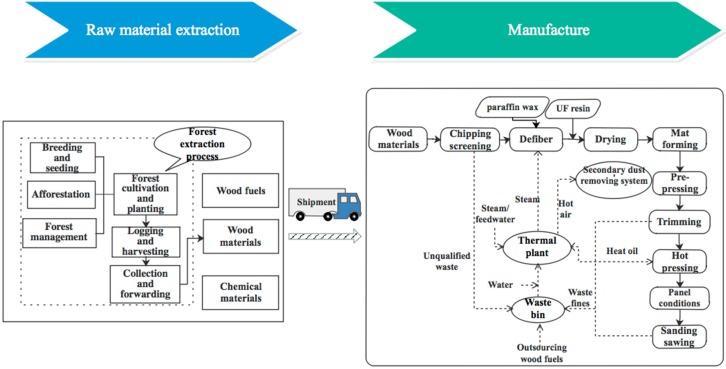
Cradle-to-gate system boundary of medium-density fiberboard (MDF) production in China.

A system boundary determines which processes shall be included in the assessments [34]. PAS 2050 and GHG Protocol allow for both cradle-to-grave and cradle-to-gate analyses. Unlike the two protocols, ISO 14067 allows the assessment of full or partial life cycle stages. Considering the uncertainty in terms of the final usage and disposal of MDF, a cradle-to-gate model was applied to enable a more precise comparison among the three protocols (Figure 1).

**Figure 2:**
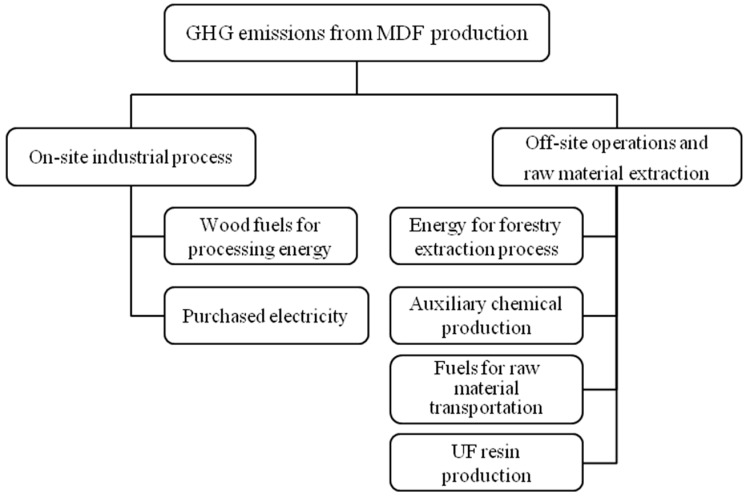
Stages considered within the cradle-to-gate system boundary in this study.

Consistent with findings by Hussain et al. [3], the cradle-to-gate system boundary of MDF production can be subdivided into two main subsystems: on-site industrial process, and off-site forest operations and raw material extraction (Figure 2).

*On-site industrial process.* All of the input amounts were reported by the surveyed company. The general steps used to produce MDF include the mechanical pulping of wood chips to fibers, blending fibers with resin and paraffin wax, drying, forming the resined material into a mat, prepressing, trimming, hot pressing, and post-processing.

*Off-site operations and raw material extraction.* The surveyed company typically used branch woods as the raw material. Among the chemicals, urea-formaldehyde (UF) resin was the most widely used material, and was self-produced by the aforementioned company. Wood fuels were obtained from the internal recycling of wood residues and purchase from other factories and forest operations. Therefore, the waste remained in the technosphere, and no allocation was conducted. Raw materials were delivered to the company through highway transportation. This subsystem includes energy for forestry extraction process, chemical production, and raw material transportation.

For cradle-to-grave assessment, this study focused on the life cycle of MDF itself, excluding the environmental impacts of its downstream industry chain. In the use stage, only carbon storage and delayed emissions were considered, because there was no energy consumption in this process. The service life (20 years) and disposal treatment pathways for MDF (scenario 0 landfill: 40%; incineration: 60%) were in line with other authors that addressed the life cycle carbon flow of wood-based panels in China [35]. A sensitivity analysis to the end-of-life disposal was conducted to evaluate the impact of different waste disposal methods on CF: scenario 1 landfill disposal (100%), and scenario 2 incineration disposal (100%).

#### 2.1.2. Cut-Off Criteria

Cut-off criteria specify the exclusions of materials, energy flow, or levels of environmental significance related to a product system [36]. By setting quantified thresholds, PAS 2050 excludes inputs lower than 1% of the anticipated total GHG emissions, and the total omissions are up to 5% [5]. No cut-off criteria exist in the GHG Protocol, because 100% completeness is necessary. Exclusions can be justified only if no data are available or an estimation proves that the process is insignificant based on mass, energy, volume, and environment. In ISO 14067, no specific criteria are available. In this study, cut-off criteria that are consistent with ISO 14044 were adopted in ISO 14067 for comparison. The application of different cut-off criteria resulted in the exclusion of several materials and fuels (Table 2). The cut-off criteria were 98.95% in PAS 2050 and 99.01% in ISO 14067.

#### 2.1.3. Capital Goods

The term capital goods is a generic label for the goods used in the life cycle of products, such as machines, equipment, and buildings [5]. PAS 2050 specifies that the GHG emissions and removals arising from the production of capital goods are excluded from the assessment. GHG Protocol also excludes GHG emissions from the production of these goods, as it excludes processes that are not directly connected to the studied product. In contrast, ISO 14067 specifies that GHG emissions can be excluded only in the absence of a significant effect on the results. The GHG emissions derived from the production of capital goods can be voluntarily included in the calculation. If capital goods were considered in the CF assessment, they would represent approximately 11.3% of the total results [37]. This study considered GHG emissions from the production of capital goods in ISO 14067, whereas PAS 2050 and GHG Protocol explicitly excluded these aspects from the boundary.

#### 2.1.4. Biogenic Carbon Removals and Emissions

Biogenic carbon is carbon derived from renewable sources, such as wood [4]. Biogenic carbon accounting is particularly important in assessing wood-based products [38]. In PAS 2050 and GHG Protocol, the impact of carbon storage is expressed as CO_2_e and deducted from the total results. ISO 14067 allows carbon storage to be separately documented. Based on the average carbon content of 52% [29], the amount of carbon embodied in 1 m^3^ of MDF is approximately 377.52 kg, which is equal to 1384.24 kg CO_2_e per m^3^ (by multiplying 44/12). Regarding emissions, the amount of CO_2_ uptake of biomass and the equivalent amount of CO_2_ emissions from the biomass at the point of complete oxidation results in zero net CO_2_ emissions. In the current study, CO_2_ emissions arising from the wood fuels are accounted as neutral and exclude from the CF assessment [39].

**Figure 3:**
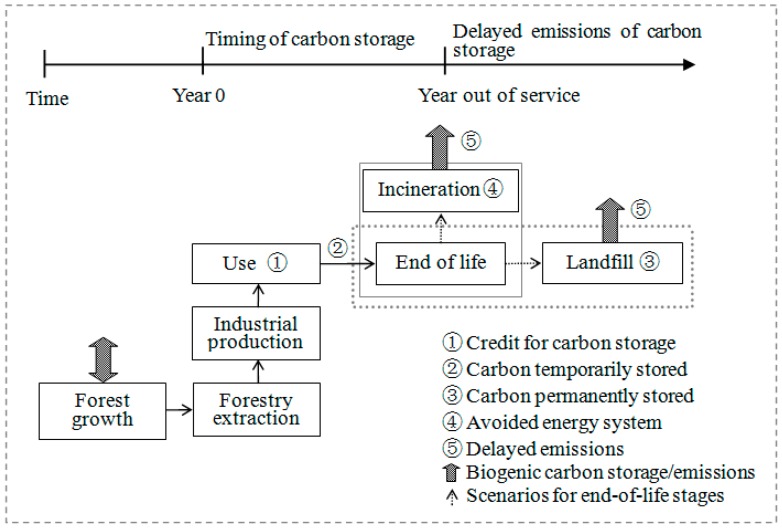
Carbon storage and delayed emissions for end-of-life treatment (incineration and landfill).

Owing to the relatively long service life of MDF, the effect of delayed emissions of carbon storage may be important to CF calculations. Among the analyzed protocols, only PAS 2050 applies a dual approach to calculate carbon storage by focusing on the effect of delaying an emission on the radiative force within the 100-year assessment period. Figure 3 shows the cradle-to-grave system boundary of MDF with two end-of-life disposal scenarios.

The incineration scenario that represents carbon storage is released after the service life, that is, the time of carbon storage is the same as that of the service life. For the landfill treatment, 98.9% of the biogenic carbon can be permanently stored, and only 1.1% of carbon is released into the atmosphere [40].

#### 2.1.5. Other System Boundary Exclusions

Emissions and removals associated with changes in land management practices are also specified in the analyzed protocols. GHG emissions from direct land-use change are all included in the three protocols analyzed. Furthermore, ISO 14067 allows GHG emissions that are derived from direct land-use change to be separately documented. Meanwhile, indirect land-use change is not a requirement in PAS 2050 and GHG Protocol. In ISO 14067, indirect land-use change should be considered when an internationally agreed procedure exists [7]. However, this study provided that forests have been sustainably managed and land use was not changed. As a result, GHG derived from land-use change was not considered. GHG emissions from indirect land-use change were also excluded due to the difficulty in calculation and prediction [15].

Other exclusions include the transport of workers to their workplace and of consumers to purchase sites, human energy inputs to the process, and animals providing transport services [41].

### 2.2. Data Collection

High-quality data are essential to provide a reliable evaluation [27]. The three protocols complied with the data quality principle specified in ISO 14044. Data are divided into two types according to their sources. Primary data came from direct measurements of MDF’s life cycle, whereas secondary data are used for inputs where primary data were unavailable [42].

The inventory analysis in this study mainly used primary data collected from the surveyed company complemented with secondary data from the national reports and the corresponding literature. The data sources used for data activity and emission factors were summarized. GHG emissions from UF resin were assumed to be those calculated by Zhang [43], because the obtained results represented the actual condition in China. GHGs emitted from the production of formaldehyde and urea were calculated using the Chinese Life Cycle Database (CLCD) [44]. The production of other secondary materials i.e., ammonium chloride and paraffin, was derived from the Ecoinvent database [45]. Other data sources for activity data and emission factors were shown in Table 3.

## 3. Results

**Figure 4:**
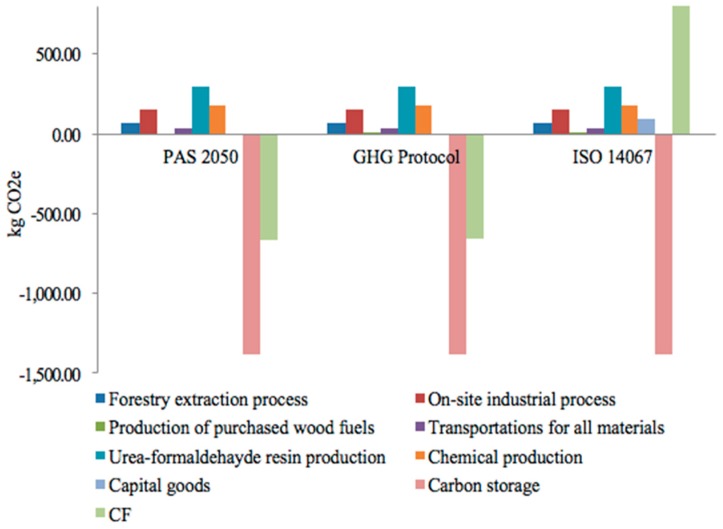
Cradle-to-gate life cycle carbon footprint of MDF quantified by PAS 2050, GHG Protocol, and ISO 14067.

Figure 4 compares the cradle-to-gate CF of calculated MDF by using the three protocols and shows the relative contributions of each life cycle stage. The CF of MDF production was −667.75, −658.42, and 816.92 kg of CO_2_e with PAS 2050, GHG Protocol, and ISO 14067, respectively. The difference mainly depended on the inclusion of carbon storage in PAS 2050 and GHG Protocol as a deduction. The only difference between PAS 2050 and GHG Protocol is the cut-off criteria. In terms of net balance, the result obtained with the ISO 14067 was −567.32 kg CO_2_e. The results obtained from this protocol were the largest not only because ISO 14067 separately reported biogenic carbon storage, but also because the protocol included GHG emissions from capital goods. As ISO 14067 required, capital goods were included in the CF result and contributed to 93.31 kg CO_2_e. Considering net CF balance, the inclusion or exclusion of capital goods was one of the main factors responsible for the differences in results. The similarities between PAS 2050 and ISO 14067 were created by cut-off criteria, because they all excluded the production of ammonium chloride, sodium hydroxide, and paraffin wax. Allocation rules exhibited a negligible contribution to the differences in the results, because of the consistent approach applied in the MDF life cycle.

**Figure 5:**
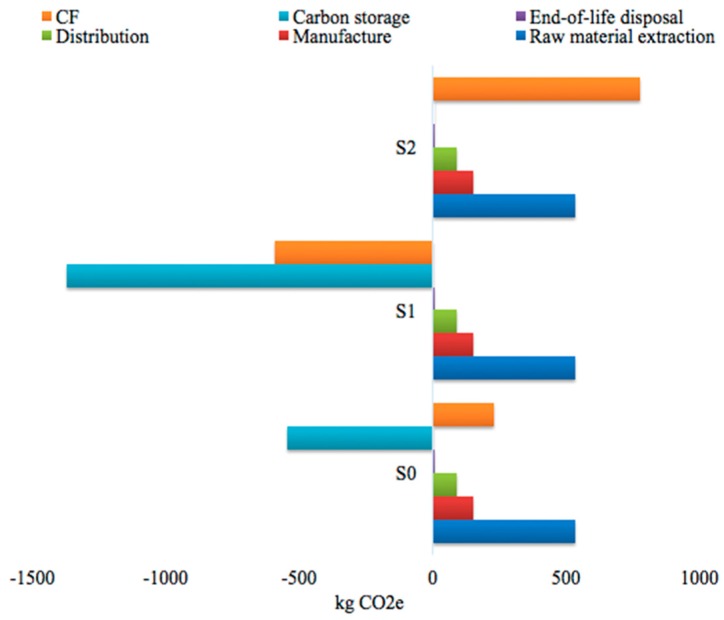
Carbon footprint of MDF quantified by PAS 2050 (cradle-to-grave assessment). S0: scenario 0; S1: scenario 1; S2: scenario 2.

In order to identify how much each life stage and emission source contributed to the CF, a cradle-to-grave assessment was also performed. Only PAS 2050 provides a method for calculating delayed emissions. Thus, this study followed the PAS 2050 to evaluate the whole life cycle of CF. Figure 4 and Figure 5 shows the relative contributions of each life cycle stage. By considering the processes that provided the most contribution to the CF, the results of all of the protocols were consistent, showing that the UF resin production was the main hotspot. UF resin contributed 36.57–41.69% to the total impact. For off-site processes, the second GHG emission contributor was chemical production. The CF values at this stage was 181.40 kg of CO_2_e per m^3^ by using GHG Protocol and 180.20 kg of CO_2_e per m^3^ by using ISO 14067 and PAS 2050, and approximately 15% were derived from urea production. Although wood provides fuels from neutral carbon in this study, the GHG emissions from the production of these materials were included [49]. In the GHG Protocol, GHG emissions from forestry extraction, the production of purchased wood fuels, and the transportation of raw materials accounted for 13.14% of the total GHG emissions. The remaining stages presented individual contributions smaller than 10% in PAS 2050 and ISO 14067.

The net GHG emissions for manufacturing were 20.77%, 20.71%, and 18.40% for PAS 2050, GHG Protocol, and ISO 14067, respectively. The main source of GHG emissions according to all of the protocols for on-site industrial processes was the electricity use, which contributed to 150.31 kg CO_2_e. If non-renewable fuels of coal and natural gas are used for heating, then the total CF is expected to be 1.05 and 1.25 times higher in natural gas and coal, respectively, than in wood fuels.

Figure 5 presents the cradle-to-grave CF obtained for the various end-of-life scenarios. The choice of the disposal pathway can considerably affect the CF of MDF. Under scenario 0 (S0), the CF result obtained with the PAS 2050 was 229.10 kg CO_2_e. For the landfill scenario (S1), accounting for the delayed emissions having less of an impact on global warming, a negative CF was calculated (−589.82 kg CO_2_e). Carbon storage in landfills was evaluated to be about −1369.01 kg CO_2_e. The scenario with incineration disposal had a higher CF than the landfill one, resulting in 238.29% more compared to the benchmark scenario. MDF with landfill disposal can act as a carbon sink. Thus, landfill would be preferable from a GHG standpoint.

## 4. Discussion

### 4.1. PAS 2050: The Most Suitable Protocol for Quantifying the CF of MDF

Discrepancies between CF methodologies cause confusion and hinder the acceptance of CF results. The comparison of the CF results highlights the importance of following a uniform protocol for quantifying GHG emissions. A key finding is that for both the production system used and the decisions taken by the person carrying out the CF result, PAS 2050 is the most suitable protocol to quantify the CF of MDF. PAS 2050 provides more specific guidance on cut-off criteria and other exclusion rules. When the CF calculation of one product is carried out using PAS 2050, the results would be lower compared to the results according to the GHG Protocol and ISO 14067 due to different cut-off criteria. PAS 2050 also adds specific requirements for collecting data in some special cases to narrow the errors. According to the Intergovernmental Panel on Climate Change (IPCC), carbon storage in wood products should be included in the national GHG inventories after the second commitment period of the Kyoto Protocol [50]. In PAS 2050, the impact of carbon storage is deducted from the total results, in compliance with the IPCC, which is favorable for recognizing the environmental benefits of forest products. In addition, only PAS 2050 proposes a weighing factor for calculating the delayed emissions of carbon storage, which is more conducive to highlighting the environmental benefits of MDF and other forest products. This protocol is widely adopted in numerous CF assessments of forest products, including softwood lumber, softwood plywood, western red cedar, western red cedar siding [51], furniture [52], ornamental plant [53], and sawn timber products [54].

GHG Protocol is based on the first version of PAS 2050 and is unlikely to result in significant differences in outcomes. Compared with PAS 2050 and GHG Protocol, ISO 14067 has limitations, including the carbon storage of products. One of its major differences from PAS 2050 is that ISO 14067 focuses on the communication of CF results [55]. Under ISO 14067, the CF results of products, data, methods, assumptions, and limitations should be reported publicly. In the communication phrase, ISO 14067 provides a standardized format that allows the transparent communication of results to the public that can take the form of a CF label, a CF external communication report, a CF performance tracking report, or a CF declaration [34].

In terms of communication, ISO 14067 allows transparent communication and can be used as the uniform standard for communicating GHG results. However, for reporting national GHG inventories and meeting GHG emission reduction targets, PAS 2050 is preferentially recommended to assess the CF of MDF and other forest products in China.

### 4.2. Implications to Fiberboard Industry

For meeting the GHG emission reduction target, some measures should be taken in the MDF industry. UF resin provided the largest contribution to the total CF in the MDF production chain. Resins are integral components and contributors to the performance of wood composites [56]. Compared with other countries, China produces 1 m^3^ of MDF, consumed 125 kg of UF resin in China, and only 44.44, 70.30, 83.30, and 85.3 kg of UF resin were required in Spain, Brazil, the USA, and Canada, respectively [25,26,27,39]. MDF manufacturing enterprises in China are mostly small and medium-scale with self-produced UF resin. Therefore, production technology may be retrograded, thereby producing increased pollution to the environment. The future of UF resin production should be toward a professional and large-scale technology.

Moreover, additional energy is consumed in transporting raw materials, given that the raw materials of companies are outsourced. In China, only approximately 14% of wood-based panel enterprises operate the management mode of “forest-panel integration”, that is, the self-sufficiency of raw wood materials by the company to independently manage the timber forest upstream. Therefore, most companies outsource to realize the supply of wood raw materials with an average transportation distance of 300 km [21]. The reduction in GHG emissions from raw material transportation can be achieved by the forest–panel integration mode of MDF companies.

### 4.3. Limitations of the Present Study

The current study compared the CF of a forest product according to three protocols. Given the fact that the inclusion (negative) or exclusion (positive) of carbon storage dominated the difference in the results, it would be more interesting to consider other products that do not store carbon for comparison. This study also excluded a few sources of GHG emissions, such as fuel consumption for operating machines and field transportation. Owing to the lack of accurate data, these exclusions could undervalue the total CF of MDF. Moreover, GHG emissions from the production of capital goods were calculated based on data from Frischknecht et al. [37], which may not represent the actual conditions in China. The detailed data regarding the CF of the capital goods of China’s wood-based panels should be collected from mills in the future.

## 5. Conclusions and Recommendations

This study discussed the outcomes of different CF protocols, using MDF as a case study. Firstly, a cradle-to-gate model was established as the basis of comparisons. Secondly, the cradle-to-grave CF was calculated considering end-of-life disposal scenarios: landfill and incineration. The main conclusions can be drawn as follows. Three CF methodologies (PAS 2050, GHG Protocol, and ISO 14067) can be applied to the MDF production of China from a cradle-to-gate life cycle. Each methodology provides a different result, although the same input data are used. The net balances of CF were −667.75, −658.42, and −567.32 kg CO_2_e kg of CO_2_e with PAS 2050, GHG protocol, and ISO 14067, respectively. As the first study to focus on the CF of the MDF industry in China, UF resin production is the major hotspot in MDF manufacturing due to backward technologies for production. Moreover, the raw materials of companies are outsourced; thus, the transportation of raw materials consumed additional energy. The mode of forest–panel integration in the MDF manufacturing enterprises in China should be established. The cradle-to-grave CF of MDF quantified by PAS 2050 was 229.10 kg CO_2_e (end-of-life: 40% landfill and 60% incineration). A negative CF was calculated with the landfill scenario, meaning that MDF may act as a carbon sink.

The discrepancy in the results can be attributed to different methodological issues, particularly the cut-off criteria, inclusion or exclusion of capital goods, and other boundary issues, and the inclusion or exclusion of biogenic carbon storage and emissions. For assessing GHG emissions in the MDF industry, discrepancies among CF methodologies cause confusion and hinder the acceptance of CF results. PAS 2050 provides more specific guidance on cut-off criteria and other exclusion rules. This protocol includes carbon storage in the CF calculations of forest products as a deduction, thereby favoring the environmental benefits of forest products compared with products that do not store carbon. For both LCA practitioners and policy-makers, PAS 2050 is preferentially recommended to assess the CF of MDF and other forest products.

## Figures and Tables

**Table 1:** An overview of key aspects specified in carbon footprint protocols: Publicly Available Specification (PAS) 2050, Greenhouse Gas Protocol Product Standard (GHG Protocol), and ISO 14067 Carbon Footprint of Products (ISO 14067).

Specifications and Requirements		PAS 2050	GHG Protocol		ISO 14067
Goals		To provide a uniform specifications for GHG emissions of goods and services	To provide detailed guidelines on accounting and reporting		To standardize the quantification process and the communication of GHG emissions
Life cycle stage included		Cradle-to-grave	Cradle-to-grave		Cradle-to-grave
					Cradle-to-gate
					Gate-to-gate
		Cradle-to-gate	Cradle-to-gate		Partial life cycle
Cut-off criteria		Exclusion based on materiality (<1%); at least 95% of the complete product life cycle must be included; no scale-up requirement to account for 100%	No cut-off criteria exist, because 100% completeness is necessary		No specific criteria available
Capital goods		Excluded	Excluded, but encouraged to be included when relevant		Excluded if they do not significantly affect the overall conclusions
Biogenic carbon	Carbon storage	Stored carbon within 100 years shall be recorded and accounted for in the CF calculations	For cradle-to-gate system, credit is given to biogenic carbon storage		If carbon storage is calculated, then it shall be separately reported but not included in the CF result
	Delayed emissions	A weighting factor is included and proposed	Shall ^a^ not be included		Shall not be included
Other exclusions	Land-use change	Specific procedure and provides default soil emissions per country	Provides guidance for determining attributable impacts		Direct land-use change shall be separately documented; indirect land-use change should be considered
	Others	Other exclusions include the transport of workers to their workplace and consumers to purchase sites, human energy inputs to the process, and animals providing transport services			
Allocation		(1) Avoiding allocation by process subdivision or system boundary expansion (2) Supplementary requirements (3) Economic allocation		(1) Avoiding allocation by process subdivision and redefining the functional unit or system expansion (2) Physical relationships (3) Economic or other allocation methods	
Global warming potential		100 years			

^a^ Shall mean recommendation.

**Table 2:** Materials and energy excluded from the cradle-to-gate assessment due to cut-off criteria.

Materials and Energy	Analyzed Protocols		
	PAS 2050	GHG Protocol ^a^	ISO 14067 ^b^
Chemicals	Ammonium chloride	None	Ammonium chloride
			Sodium hydroxide
			Herbicide
	Sodium hydroxide		Paraffin wax
	Paraffin wax		K fertilizer
Energy	Energy for seedling cultivation	None	Gasoline for harvesting
	Energy for tree plantation		
	Energy for total thinning		

^a^ Under this protocol, the present study considered all of the unit processes significant to conduct a full life cycle inventory. ^b^ Under this protocol, a flow of less than 1% of the cumulative mass or energy was excluded from the boundary.

**Table 3:** Data sources for activity data and emission factors used to quantify the carbon footprint of medium-density fiberboard (MDF). UF: urea-formaldehyde.

Stages	Data Source	
	Activity Data	Emission Factor
On-site industrial process		
Wood fuels for energy	Surveyed company	Ecoinvent 3.0 [45]
Electricity	Surveyed company	NDRC ^a^
Off-site process		
Capital goods	Frischknecht et al. [37]	Frischknecht et al. [37]
Energy for forestry extraction		
Petroleum	Lun et al. [35]	IPCC [19]
Gasoline	Lun et al. [35]	IPCC [19]
Diesel	Lun et al. [35]	IPCC [19]
Electricity	Lun et al. [35]	NDRC
Fertilizer and biocide	Lun et al. [35]	West et al. [46]
Chemical production		
Formaldehyde	Surveyed company	CLCD [44]
Urea	Surveyed company	CLCD [44]
Ammonium chloride	Surveyed company	Ecoinvent 3.0 [45]
Sodium hydroxide	Surveyed company	ELCD [47]
Paraffin wax	Surveyed company	Ecoinvent 3.0 [45]
UF resin production	Surveyed company	Zhang [43]
Transports		
Distances	Surveyed company and national reports ^b^	CLCD [44]
Fossil fuels	Surveyed company	CLCD [44]
End-of-life disposal	Demertzi et al. [48]	
Energy for landfill		
Electricity		NDRC
Diesel		IPCC [19]
Energy for incineration	Demertzi et al. [48]	
Electricity		NDRC
Diesel		IPCC [19]
Natural gas		IPCC [19]

^a^ National Development and Reform Commission, 2016. ^b^ Data obtained as average haul distances of goods in China, 2016. Referring to 2016 China Statistical Yearbook. IPCC: Intergovernmental Panel on Climate Change; CLCD: Chinese Life Cycle Database; ELCD: European Reference Life Cycle Database.

## Nomenclature

BSI	British Standards Institution
CF	carbon footprint
CLCD	Chinese Life Cycle Database
CO_2_	carbon dioxide
CO_2_e	carbon dioxide equivalent
GHG	greenhouse gas
GHG	Protocol: GHG Protocol Product Standard
GWP	global warming potential
IPCC	Intergovernmental Panel on Climate Change
ISO	International Organization for Standardization
ISO 14067	ISO 14067 Carbon Footprint of Products
MDF	medium–density fiberboard
PAS 2050	Publicly Available Specification 2050
m^3^	cubic meter
UF resin	urea-formaldehyde resin
WBCSD	World Business Council for Sustainable Development
WSI	World Resources Institute
